# Supplementary material for: Conflict, caregiver violence and gendered parenting: A cross-sectional study among adolescent girls and young women participating in a girls’ empowerment programme in Myanmar
Source: PLOS Glob Public Health. 2025 Oct 24;5(10):e0004737. doi: 10.1371/journal.pgph.0004737 (PMC12551872; doi:10.1371/journal.pgph.0004737)
Supplement: Attachment — Submitted filename: Response to reviewers.docx [file pgph.0004737.s003.docx]

| Reviewer 1 |  |
| --- | --- |
| 1. The methods section should specify the Country, not only the regions and state | Thank you for this comment. To clarify, in the first sentence of the Methods section, we have stated that the country is Myanmar, so did not repeat this when noting the regions/states. If you prefer us to repeat this information, please let us know. |
| 2. The conclusion could be more specific by explicitly stating the study’s implications. | Thank you for this helpful suggestion, the following paragraph is now added to the conclusion:  “Interventions should ensure the physical safety and psychological wellbeing of girls and young women, whilst also engaging boys and young men to ensure they are not reflecting their parents' or society’s negative gendered attitudes. These findings highlight the need for adaptable, multi-sectoral interventions that reflect the complexity of conflict-affected settings and girls’ well-being, which require activities related to violence prevention, mental health support, empowerment, and the shifting of harmful gendered norms within families and communities.” |
| 3. Grammar and sentence structure should be revised throughout the paper for clarity and readability. | Thank you for this input – we have since given the manuscript a thorough re-read and improved some of the grammar and sentence structures – please see tracked changes throughout the updated document. |
| 4. The clarity of sentences should be revisited to ensure grammatical accuracy. | Thank you, as per our response to comment 3, please see tracked changes within the document. We hope we have addressed your concerns. |
| 5. Why there is only 715 from total of 731 respondents answered the questions on their experience of parent/caregiver-perpetrated psychological and physical violence, while actually there is an answer option of: Never, meaning not all of them respond to this question are the victim of violence? | Thank you for requesting this clarification. All respondents (n=731) were offered the option whether or not to respond to this section of the survey. Ultimately, of the total respondent group (n=731), 715 respondents opted to answer the full violence question set, and 16 chose to skip either some or all of the violence questions. For those who opted to answer ‘yes’ to the set of questions (n=715), they had the option of reporting that they “never experienced violence”, which was an option in the answer set. But to clarify, answering “never experienced violence” is not the same as choosing not to answer the questions. You are correct to conclude that not all respondents had experienced violence, which is reflected in those who answered “never” to the violence questions. |
| 6. Add some references to enhance the literature review:   Caregiver parenting and gender attitudes: Associations with violence against adolescent girls in South Kivu, Democratic Republic of Congo  Gendered and transactional relationships: Children's experiences of being parented and perspectives on their relationships with their parents in Uganda | Thank you for these suggestions, the references are interesting and very relevant to this study. I have now added the first reference with (Falb et al., 2017) with citation to the background of gendered parenting, the addition reads: “Evidence from conflict-affected settings, such as the Democratic Republic of Congo, suggests that caregiver’s gender-equitable attitudes was associated with reduced risk of sexual abuse among adolescent girls, and reduced likelihoods of girls’ acceptance of IPV “  As for the second reference (Siu et al., 2025), I have now added the following summary with citation to the first paragraph of the gendered parenting section of the introduction: “For example, qualitative research from Uganda found that boys experienced greater freedom to play in their free time when compared to girls, who instead reported spending their free time learning gender-specific roles such as cooking” |
| Reviewer 2 |  |
| 1. This is a very timely and needed study carefully conducted in an active conflict situation. The authors and their counterparts should be applauded for conducting this study in such a fragile situation. It is a well-written paper in excellent academic English language. | Thank you so much for your feedback and thoughtful review - we really appreciate you taking the time to provide your comments. |
| 2. Title: I am a bit concerned about this components of the title: A cross-sectional study among adolescent girls and young women in Myanmar    a. As of now, it is implied that the study is representative of Myanmar. However, from methodology, it is clear that the sample frame is adolescent girls and young women who are participating in the circle programme, not the total adolescent and girl population of Myanmar.    b. Suggest indicating this in the title. Ex: A cross-sectional study among adolescent girls and young women participating in a weekly girls empowerment programme in Myanmar    c. This is especially true, as the study attempts to provide a population proportion with a confidence interval, however. The sampling method, as I understand, is not a probabilistic sampling method. While I totally appreciate that would be not feasible, authors need to be careful that they are not over-generalizing their sample to the total of the adolescents and girls in Myanmar. | Thank you for this helpful feedback, we agree that the title has potentially misleading implications.. Per your suggestion, we updated the title:  “Conflict, caregiver violence and gendered parenting: A cross-sectional study among adolescent girls and young women participating in a girls’ empowerment programme in Myanmar” |
| 3. It would be helpful to indicate how similar and different are girls and women in circle sites? How long has these circles been running? Has there been any awareness raising or empowerment that happened in these circles before the study? In such cases, the sampled population is likely to be different from the average girls and women population, right? | Thank you for this comment. We have now added some more details about the *Circles* programme to the “Study design and participants” section, which now reads:  “We conducted this study in collaboration with Myanmar-based NGO, Girl Determined (GD). GD supports girls and young women aged 11–25 years from low-income rural and urban settings across Myanmar. The two-year core programme, Circles, is an in-person weekly girls empowerment programme, facilitated by women hired and trained from local communities. Circles covers topics such as: self-confidence, assertiveness, ethical and religious discrimination, trafficking and safe migration practices, stress management, relationships (both romantic and friendships), drug awareness, goal-setting and sexual and reproductive health education. GD recruits hard-to-reach girls through sites such as camps for internally displaced people, churches, Buddhist monastic schools and dormitories for girls from rural areas. Therefore, all girls enrolled in Circles generally represent girls from the lowest socio-economic quintiles in the regions of Myanmar that GD is active in. Due to GDs targeted outreach to nunneries and boarding houses, our sample included a high proportion of girls who were living away from home and enrolled in education, as these institutions provide education and accommodation to girls from low-income communities across Myanmar. At the time of survey, each participant was enrolled in GD’s Circles programme and enrolment occurs at the site-level, for example, all girls aged 10-18 years who are residing at a selected boarding house will be invited to join. For more details about GD’s work, please see Girl Determined.”  We have also amended the following sentence in the “Benefits and limitations” section of the discussion to capture your point about the representativeness of the sample and highlight prior training that the girls have received through Circles. This now reads:  “Participants were invited who were current beneficiaries of GD’s empowerment programme, which limits the generalisability of findings to girls outside this programme. It should also be noted that the participants of this study had already received training and education on topics such as gender equality and violence, which may have influenced their responses to questions around these topics.” |
| 4. Also please see my comment about a map demonstrating sampling sites so that the reader gets a clear idea about the generalizability of the sample. | Thank you, please see our response to comment 8 which addresses this. |
| 5. It would be good to mention about the current systems or lack thereof for the prevention or management of parent/caregiver violence in Myanmar, including support networks. Are there any systems that cater to the parents and caregivers? Unlikely, but I think it is essential to highlight this. I was wondering if you should indicate health/mental health and psychosocial support systems (or lack thereof) in your conceptual framework. | Thank you for this very helpful input. We have now added some more information about this. First, we added to the background section on VAC in Myanmar, specifically the paragraph which now reads:  “Despite this, formal systems for preventing or responding to VAC in Myanmar are scarce and policy legislation around physical discipline of children remains ambiguous (41). Although Myanmar adopted a new Child Rights Law in 2019 prohibiting all forms of physical punishment of children, such acts are still widely accepted in the home, day care, schools and alternative care settings (41,42). Ultimately, efforts to prevent or address VAC in Myanmar remain limited, and research is needed on how the ongoing conflict may influence the prevalence of VAC, especially among adolescent girls, who face intersecting risks related to their age and gender.”  And also to the conceptual framework description paragraph, which now concludes with the text:  “These health and wellbeing outcomes will be at greater risk especially due to the continued political instability, which is blocking the already-limited mental health and psychosocial support services, especially in the rural and conflict-affected areas (55).”  We had added additional new references throughout to support these statements (refs 41, 42 and 55). We have also now edited the figure 1 (conceptual framework diagram) to include the breakdown in health services – this edit can be found in the first line of the “Armed conflict & instability” component, which now reads: “Breakdown of rule of law, health & social services”. We have also edited figure 1’s format, colours and text slightly to make it clearer. |
| 6. Out of the three major components of the conceptual framework, all except Gender Inequality and Societal Norms have nicely been addressed in the sections prior to that. Suggest adding a subsection titled Gender Inequality and Societal Norms, so that it would be cognitively easier for the reader to direct the reader to the conceptual framework. | Thank you for this very helpful observation. You are right that we missed an introduction to this topic in the background section. We have now added a paragraph titled “Gender inequality and societal norms”, which reads:  “Gender inequality and societal norms  Gender inequality is prevalent across Myanmar, stemming from historically patriarchal traditions and societal norms that shape the expectations of girls and women from a young age. A key example is the concept of *hpon*, a widespread notion of male superiority over women under Theravada Buddhism in Myanmar (55). *Hpon* perpetuates beliefs around women’s impurity, positioning this impurity as a direct threat to men’s *hpon*, which has contributed to women being excluded from leadership roles and restricted from certain religious spaces (55,56). Similar gendered hierarchies exist among other religious and ethnic communities, and research has shown that women from minority backgrounds are disproportionately impacted by these inequalities (56). Women and girls across the country risk stigma and consequences if they fail to uphold the gendered expectations of obedience and modesty that are expected of them, and adolescent girls are especially impacted by these gendered norms due to their young age (55–57). For example, from an early age, many girls are socialised to take on household responsibilities and gendered roles within the family to be considered a “good daughter”, and—despite greater emphasis on girls’ education in recent years—their schooling is still widely considered to be less important than boys’, especially in financially insecure settings (58). Furthermore, girls must navigate entrenched taboos around their sexuality and reproductive health and can face stigma following experiences of gender-based violence, restricting their ability to access information and support and putting their health and wellbeing at risk. These dynamics are further heightened during times of conflict, economic instability and displacement, which simultaneously exacerbate risk to girls’ health whilst also weakening the systems that may otherwise protect them (58–60).” |
| 7. Does the purple colour in the conceptual framework have any specific meaning or relationship? If so, it would be good to elaborate, as a footnote | The use of purple in Figure 1 is seeks to represent how gender inequality and societal norms (red) intersect with armed conflict and political instability (blue), to indicate how when these components intersect, they give rise to gendered parenting practices and violence, shown in purple to reflect their compounded influence on adolescent girls’ mental health and wellbeing.  Thank you for pointing out this ambiguity – we have now amended the figure caption as follows: “Figure 1: Conceptual framework for our analysis, summarising hypothesised links between armed conflict, gender inequality, gendered parenting practices and parent/caregiver perpetrated violence among adolescent girls and young women. The purple boxes represent domains shaped by both the armed conflict and gender inequality, to reflect their compounding influence.” |
| 8. An average reader might not be familiar with the regions of Myanmar. It would be good to demonstrate the sampling regions and sites of them. This will also help the reader to better understand the sampling frame and mechanism, as well as the generalizability of the study to Myanmar. Just a suggestion. | Thank you for this suggestion. I have modified the following map taken from Wikipedia - which I believe can be published with the following footnote/caption: (Adapted from Aotearoa, Own work, CC BY-SA 3.0 https://commons.wikimedia.org/w/index.php?curid =1234889  Modifications: red boxes added; this adapted work is licensed under the same terms: CC BY-SA 3.0 <https://creativecommons.org/licenses/by-sa/3.0/>)  Please find the map at the bottom of this page.  Note to Editor: please can you confirm whether this caption is sufficient for the map to be published in the manuscript? |
| 9. Figure 3: It took me a while to understand what the left and right % values mean (26%,.... On the left and 74%,.... On the right). I understood that left is % for girls and right is the % for boys (brothers), right? Better to add a female or male symbol or a sign above the percentage to help the reader understand this figure easily. | Thank you for your comment. Actually, the right and left of the graph are displaying the level of agreement (on the right) compared to the level of disagreement (on the left) among girls only– boys were not consulted. Thank you for highlighting that this is unclear. We have now amended the figure caption to explain the graph a little better, which now reads:  “Figure 3: Percentage of participants (n=514) who agreed or disagreed with statements comparing their experiences of gendered parenting to how they perceive their brother(s) to be parented. Agreement (right-hand side) and disagreement (left-hand side) are shown across four response categories: strongly disagree, slightly disagree, slightly agree, strongly agree.” |
| 10. Table 4 - Suggest reformatting as a standard multiple regression table, for example, as for the following format.     a. As of now it is a bit complex to understand with the first column title of exposure and the column titled reference group     b. Also it is suggested to have a univariate analysis table prior to the multiple logistic regression table.     c. Consider adding comparison table for exposed and unexposed before the multiple logistic regression table. | Thank you for this thoughtful suggestion. We agree that univariate and descriptive comparison tables can be valuable in some contexts. However, in this instance, we chose to present only the adjusted regression results to avoid redundancy, as the variables included in the model were theory-driven and selected based on prior literature and conceptual relevance rather than data-driven significance testing. Additionally, including separate univariate or exposed/unexposed comparison tables would substantially increase the length of the manuscript without offering additional insight beyond what is already captured in the multivariate models and described in the text. To improve clarity, we have reformatted the regression table to better align with standard conventions, as per your helpful suggestion. |
| 11. It is worth discussing the role of qualitative methods in improving the depth of understanding a topic like this. | Thank you for raising this, we have now added this short paragraph to the benefits and limitations section which discusses this:  “Furthermore, while this study offers important insights into the quantitative associations between key variables, the complexity of adolescent girls’ experiences highlights the need for further complementary qualitative research to better understand the nuances behind these intersecting risk factors. While outside the scope of this study, qualitative data from parents and caregivers would offer a further understanding of gendered parenting practices and the use of violence within the home, including why these behaviours may be exacerbated during periods of instability and uncertainty. Such research could also help to identify contextually appropriate targets for future interventions to protect adolescent girls from these risk factors.” |
| 12. Conclusions: It may be good to discuss how the Circles could be used to address this issue. Can the program reach out to parents? Can awareness raising be conducted to the parents through the circle program? I was wondering what would caregivers think about these findings? Can we use these findings to advocate for the prevention of violence by caregivers? | Thank you for raising this important point – We have now added the following sentences to the conclusion:  “Programmes such as Circles offer a valuable opportunity to support girls and young women, not only directly, but also through targeted outreach to parents and caregivers. This study’s findings may serve as a useful advocacy tool to encourage caregivers to reflect on their parenting practices and to inform intervention strategies that promote safer and more gender-equitable home environments.” |
